# Child Care as an Untapped Setting for Obesity Prevention: State Child Care Licensing Regulations Related to Nutrition, Physical Activity, and Media Use for Preschool-Aged Children in the United States

**Published:** 2008-12-15

**Authors:** Karen M. Kaphingst, Mary Story

**Affiliations:** Healthy Eating Research, Division of Epidemiology and Community Health, School of Public Health, University of Minnesota; University of Minnesota, Minneapolis, Minnesota

## Abstract

**Introduction:**

Child care is a potential setting for obesity prevention; 8.6 million preschool-aged children participated in child care in 2001. Each US state creates and enforces its own child care licensing regulations. We analyzed obesity-related child care licensing regulations of US states.

**Methods:**

We downloaded state licensing regulations for children in child care centers (CCCs), small family child care homes (SFHs), and large family or group child care homes (LFGHs) in each state and the District of Columbia (collectively referred to as "states") in 2006 from national and state Web sites. We conducted a quantitative content analysis to identify 13 coding dimensions related to nutrition, physical activity, and media use.

**Results:**

We found variability among and within states. CCCs were the most heavily regulated and had the most specific regulations, followed by LFGHs. SFHs had the fewest and most general regulations. Just 2 states, Michigan and West Virginia, specified that CCC menus should be consistent with the *Dietary Guidelines for Americans*. Only 12 states had regulations that limited foods of low nutritional value in CCCs. Thirty-six states required that children have daily outdoor activity time in CCCs; only 9 states set specific minimum lengths of time that children should be outdoors each day. Eight states set quantified time limits on screen time per day or per week in SFHs.

**Conclusion:**

Opportunities exist for strengthening state licensing regulations to prevent childhood obesity. The increasing prevalence of childhood obesity underscores the urgency for state policy efforts to create child care environments that foster healthful eating and participation in physical activity.

## Introduction

Approximately 1 of every 4 children aged 2 to 5 years in the United States has a high (≥85th percentile for age) body mass index (BMI) (1). Overweight and obesity are associated with adverse health consequences, such as type 2 diabetes, hypertension, hyperlipidemia, sleep apnea, and psychosocial issues (2), even in childhood, and overweight children are more likely to become obese adults (3). Overweight and obesity are among the most challenging health issues of our time (4).

Because more children are in child care programs today than ever before, the potential for successfully addressing childhood obesity in the child care setting is great. In 2002, 60% of mothers with preschool-aged children (younger than 6 years) were employed; 70% of those mothers worked full-time, and 30% worked part-time (5). In 2001, 73% of preschool children aged 3 to 5 years (8.6 million preschoolers) participated in at least 1 weekly nonparental care arrangement (6). Nationwide, nearly 60% of children aged 3 to 4 years with employed mothers participate in center-based care (45%) or child care in a family home (14%) as their primary child care arrangement, and 18% are cared for by a parent, 17% are cared for by another relative, and 6% are cared for by a nanny or babysitter (7). Forty-one percent of preschool-aged children are in child care for 35 or more hours per week (8).

The child care licensing office for the 50 US states and the District of Columbia (DC) (collectively referred to as "states") creates and enforces specific minimum health and safety requirements that regulated providers must meet to operate legally (9). Almost all child care centers are regulated in some way, but state regulations for small and large family child care homes vary (10). Localities and individual child care facilities can adopt additional policies and practices to augment state policies, but they must also meet state requirements.

Early childhood is an important period for developing dietary and physical activity behaviors (11), and practices of child care facilities can improve children’s dietary intake, physical activity levels, and energy balance. However, little is known about state policies related to food intake and physical activity in child care settings. We examined state-level child care licensing regulations for all states related to nutrition, physical activity, and media use for preschool-aged children (typically defined as children aged 3 to 5 years) in 3 types of child care settings: 1) child care centers (CCCs), 2) small family child care homes (SFHs), and 3) large family or group child care homes (LFGHs). To date, there has been little examination of state-level child care licensing regulations related to nutrition, physical activity, and media use.

## Methods

### Sample

We downloaded child care licensing regulations for preschool-aged children from the Internet and coded them from January through April 2006 for all states. We calculated percentages by using 51 as the denominator. Only CCC, LFGH, and SFH care settings with mandatory licensing regulations were included in this analysis. Head Start regulations were not included because care provided at Head Start sites is subject to the federal Head Start Program Performance Standards (12).

### Coding dimensions

We developed standardized coding dimensions to assess licensing regulations related to nutrition, physical activity, and media use (Table 1). We examined state licensing regulation documents and coded them as either having or not having the dimensions or characteristics of the 13 regulations included in this analysis. We selected coding dimensions on the basis of obesity-related themes that emerged from the data and on the basis of scientific literature and national standards and recommendations, especially the *Dietary Guidelines for Americans, 2005* (13), *National Health and Safety Performance Standards: Guidelines for Out-of-Home Child Care* (14), and a 2005 position paper of the American Dietetic Association (15).

### Coding procedure

We conducted a quantitative content analysis to characterize the percentage of states with certain features in their licensing regulations. We analyzed regulations to determine whether the 13 coding dimensions related to nutrition, physical activity, and media use were included. To ensure that an exhaustive and current set of documents was obtained for each state, we downloaded each state’s regulatory documents from 2 sources: 1) a database maintained by the National Resource Center for Health and Safety in Child Care and Early Education, which is funded by the US Department of Health and Human Services (16); and 2) child care licensing Web sites from each state government. We saved all licensing regulation documents and screen shots of each state’s relevant Web pages on child care licensing as portable document format (PDF) files. These Web pages contained the definitions and descriptions of the child care settings provided by each state.

We examined each document for predefined key words and phrases related to the 13 coding dimensions. We examined each state's licensing regulations separately for CCCs, LFGHs, and SFHs, as applicable (eg, some states do not regulate SFHs, some states do not use the LFGH category). Licensing regulations relevant to this analysis were most often found in sections entitled Meals and Snacks, Nutrition, Food Service, Program, Equipment, Activities, and Outdoor Area/Activities. After reading through the document, paying particular attention to the these sections, we performed a word search function within Adobe Acrobat for predefined words and phrases related to any coding dimensions that had not been found in the document (eg, *Dietary Guidelines*, vending, large muscle, vigorous, outdoor, television).

A second trained coder independently coded a randomly selected sample of 30% of the state regulations (ie, regulations of 15 states for each of the 3 care settings). The second coder was trained by reviewing coding dimensions with the primary coder (K.M.K.) and then independently coding test regulations until consensus was achieved. High levels of intercoder reliability were achieved between the 2 coders. Values for Cohen's κ coefficient were calculated using SAS version 9.1 (SAS Institute Inc, Cary, North Carolina) for all 13 coding dimension variables. Complete agreement (κ = 1.0) was found between coders for 10 of the variables. Acceptable agreement, beyond the level expected by chance, was found between coders for the 3 remaining variables (κ = 0.79, κ = 0.81, and κ = 0.91).

## Results

Regulations varied considerably from state to state, and within each state, regulations often varied for different types of child care settings (Table 2). Overall, CCCs were the most heavily regulated, followed by LFGHs. SFHs had the fewest regulations. CCCs had the most specific and strongest regulations. Many states exempted SFHs from licensing requirements, opting instead to allow voluntary registration.

### Nutrition

Nutrition regulations varied among states. The most common regulation was for child care providers to follow the federal Child and Adult Care Food Program (CACFP) (17) or similar meal pattern requirements. Twenty-nine states require this regulation for CCCs, 24 states require it for LFGHs, and 20 states require it for SFHs.

Just 2 states, Michigan and West Virginia, specify that CCC menus should be consistent with the *Dietary Guidelines for Americans*. Nineteen states' CCC licensing regulations specify the number of meals and snacks to be offered by defined, comprehensive increments of time in care, up to a full day. Eleven states outline such requirements for LFGHs, and 9 do so for SFHs. An alternative approach adopted by some states is to require a specific proportion of daily nutrition needs per meal or by length of time in care (14 states required this for CCCs, 8 for LFGHs, and 6 for SFHs).

Twelve states had regulations prohibiting or limiting specified foods of low nutritional value in CCCs, as did 7 states for LFGHs and 4 states for SFHs. No states provided specific nutrition standards or criteria, such as limits on total or saturated fat or maximum number of calories. Four states, all located in the southeastern United States, regulate vending machines in at least 1 of the 3 child care settings. Alabama, Georgia, and Louisiana prohibit vending machines in areas used by children, and Mississippi requires foods sold in vending machines to meet overall nutrition standards. Georgia and Mississippi apply these same regulations, respectively, to the LFGH setting. The only other state to regulate vending machines is Arkansas, whose regulation applies only to school-aged children.

### Physical activity

For physical activity, states were most likely to require large muscle or gross motor and daily outdoor activity time for children in care. Thirty-nine states required that the program of activities in CCCs engage children in large muscle or gross motor activities or provide activities or equipment that focus on large muscle or gross motor development. Half (n = 25) of the states required this for LFGHs, and 19 states required this for SFHs.

Roughly three-fourths of the states (n = 36) required that children have daily outdoor activity time in CCCs, as did approximately half of the states for LFGHs (n = 27) and SFHs (n = 24). However, only 9 states set specific minimum lengths of time that children should be outdoors each day. Of these states, most required at least 1 hour each day. CCC regulations were most likely to include a quantified minimum amount of time. DC and Mississippi require the greatest amount of outdoor time for children, specifying that children in a full-day program shall have at least 2 hours of outdoor activity per day and that children in a part-day program shall have at least 30 minutes per day.

Licensing regulations in 10 states specify that children shall be engaged in vigorous play or physical activity (we coded for any inclusion of the term *vigorous*). Eight states require vigorous play or physical activity for CCCs, 4 require it for LFGHs, and 2 require it for SFHs. Alaska and Hawaii apply this regulation to all 3 child care settings. No states used the term *moderate* to describe the appropriate level of physical activity.

Two states, Alaska and Massachusetts, quantify the amount of time that children should be engaged in physical activity. Alaska's regulations mandate that "opportunities be provided for a minimum of 20 minutes of vigorous physical activity for every 3 hours the facility is open between the hours of 7:00 am and 7:00 pm." Massachusetts's regulations call for "thirty minutes of physical activity every day." Alaska's regulation pertains to all types of child care settings; Massachusetts' regulation applies only to SFHs and LFGHs.

### Media use

States that addressed media use in child care settings included a range of media in their licensing regulations. The most frequently mentioned were television, videos, video games, and computers. Approximately one-fourth (n = 14) of the states address, in a nonquantified manner, the appropriate inclusion of media in the child care program of activities in at least 1 care setting. Examples include specifying that media use shall meet a defined educational objective, be used only as an enhancement to the daily program, not be used as a substitute for planned activity, or be "limited" or "not excessive." Many states with media regulations specify that alternative activities should be available for children who do not wish to participate in media viewing.

Ten states set quantified time limits on screen time per day or per week in at least 1 care setting. Seven states apply this regulation to CCCs, 9 states to LFGHs, and 8 states to SFHs. Most states set limits for screen time at a maximum of 2 hours of use per day, and most applied the same time limit for CCCs, LFGHs, and SFHs. Alaska limits screen time for media exposure to a cumulative sum of 1.5 hours per day but allows an additional 2 hours per day for "computer learning activities." Maine and New Mexico limit screen time to 1 hour per day. Only Vermont sets a maximum number of hours per week in some settings; in CCCs and LFGHs, media use is limited to 5 hours per week. Vermont's SFHs are allowed a maximum of 2 hours per day.

## Discussion

The findings of our study indicate that opportunities exist for strengthening state licensing regulations to prevent childhood obesity in the child care setting.

### Nutrition

Children potentially obtain a large portion of their caloric intake in the child care setting (17,18). Several national organizations recommend that child care facilities follow CACFP guidelines for children in care. CACFP provides federal funds for meals and snacks served to children in eligible, licensed, public or private nonprofit child care centers; for-profit child care centers serving 25% or more low-income children; after-school programs; Head Start programs; and other institutions that are licensed or approved to provide day care services. Child care homes can participate in CACFP only if they have a recognized sponsor to serve as an intermediary between them and the responsible state agency (17). We found that many states rely on specified meal pattern requirements, such as those found in CACFP, to provide good nutrition in the child care setting. However, only approximately 60% of states specify meal pattern requirements for CCCs, approximately 50% do so for LFGHs, and approximately 40% do so for SFHs. One noteworthy finding of our analysis is that no state licensing regulations mandate that child care facilities meet specific nutrient-based standards. Only 2 states instruct at least 1 of the 3 child care settings examined to comply with the *Dietary Guidelines for Americans* (13).

Little research has explored the nutritional quality of foods and beverages provided in the child care setting, and our findings suggest that there may be cause for concern in this area. Existing studies have largely examined nutritional quality of meals served in facilities formally enrolled in CACFP. The most recent national survey describing the nutrient content of meals and snacks in both child care centers and day care homes participating in CACFP was conducted in 1995 but included only children aged 5 years and older (18). This study found that lunches offered to children did not meet the *Dietary Guidelines* for total fat or saturated fat; 50% served lunches with 35% or more calories from fat, and less than 5% of lunches met the recommendation for the percentage of energy from saturated fat (<10%). On average, 90% of breakfasts and 87% of lunches complied with CACFP meal pattern requirements. The food component most often missing from meals was fruits and vegetables (18). Only 42% of daily CACFP breakfast menus, 37% of lunch menus, and 25% of snack menus offered fresh fruit. Vegetables were rarely offered for morning or afternoon snacks (<5% of snacks included vegetables) (18). A 1999 national study of CACFP meals and snacks conducted in tier 2 child care homes (neither located in a low-income area nor operated by a low-income provider) examined meals and snacks offered to children aged 2 years and older (19). Meals and snacks provided, on average, more than two-thirds of the recommended dietary allowance for calories and key nutrients. Mean saturated fat content exceeded national recommendations. Approximately one-third of the morning snacks (31%) and afternoon snacks (28%) included any fresh, canned, or dried fruit. Only 2.5% of the afternoon snacks included vegetables.

A few smaller-scale studies have evaluated the menus and mealtimes in child care settings and also show cause for concern (20-24). Data on CACFP-participating child care centers in 7 states showed that meal patterns were inconsistent with the *Dietary Guidelines* regarding fat, sodium, fruits and vegetables, and serving a variety of foods (20). Menus were high in fat and seldom provided recommended servings of vegetables. A study conducted in 9 Texas child care centers compared dietary intakes of children attending child care with the recommendations of the Food Guide Pyramid for Young Children. Results indicated that children aged 3 years consumed sufficient fruits and meat/alternates but not sufficient vegetables, grains, or dairy to meet two-thirds of Food Guide Pyramid recommendations and that children aged 4 to 5 years consumed only sufficient dairy (22). These studies suggest that the dietary quality of foods provided in child care settings may need improvement. Strengthening state regulations could play a critical role in improving children's nutrition in child care settings.

### Physical activity

Physical activity positively affects overall health and helps prevent obesity (13,25). We found that physical activity licensing regulations varied markedly among and within states. Most states require CCCs to facilitate large muscle or gross motor activity and to ensure that children have outdoor activity time each day, both of which are *National Health and Safety Performance Standards* (14). However, a much smaller proportion of states have adopted more rigorous and specific regulations related to physical activity that are tied to national recommendations.

National recommendations indicate that children older than 2 years should engage in at least 60 minutes of moderate-intensity or vigorous-intensity physical activity on most, preferably all, days of the week for the maintenance of good health and fitness and for healthy weight gain during growth (13,26). The National Association for Sport and Physical Education recommends that preschoolers accumulate at least 60 minutes daily of structured physical activity and at least 60 minutes per day of unstructured physical activity and should not be sedentary for more than 60 minutes at a time except when sleeping (27). To help meet daily physical activity recommendations for preschoolers, experts have recommended that planned physical activity should be incorporated into the daily preschool schedule. Structured activity sessions should be short, approximately 15 to 20 minutes long, and should emphasize varied movements (27,28). Our analysis found that just 2 states specify a required number of minutes of physical activity per day or based on the length of time that a child is in care. Only 9 states quantify a minimum length of outdoor time each day.

Relatively little is known about the physical activity levels of preschool-aged children in child care, but preschool-aged children in child care settings may not be meeting national recommendations for daily physical activity (29). Research emphasizes the importance of the child care setting as a predictor of physical activity levels for children in this age group (30,31). Children's physical activity levels varied widely in a study of 9 South Carolina preschools (31); mean minutes of moderate-intensity to vigorous-intensity physical activity, as measured objectively using accelerometers, varied from 4.4 to 10.2 minutes per hour. The preschool attended accounted for a substantial proportion of the variability in child physical activity. In another study, the relationship between the child care environment and physical activity behavior of preschool children attending 20 child care centers was examined (32). Children in centers with environments supportive of physical activity achieved more moderate-intensity to vigorous-intensity physical activity, spent less time in sedentary activities, and had higher mean physical activity levels than children in centers with environments less supportive of physical activity. Aspects of the environment related to physical activity behavior included active opportunities, portable play equipment, fixed play equipment, and physical activity training and education of staff. These studies suggest that policies and practices can increase the overall physical activity levels of young children.

### Media use

Children in the United States are exposed to media use from their earliest years. A 2003 study from the Kaiser Family Foundation reported that children aged 6 years and younger spend an average of 2 hours per day with screen media, mostly watching television and videos (33). The American Academy of Pediatrics recommends that television time should be limited to no more than 1 to 2 hours of quality programming per day for children over 2 years of age (34). The *Dietary Guidelines for Americans* state that it is "important during leisure time to limit sedentary behaviors, such as television watching and video viewing, and replace them with activities requiring more movement" (13). Research has found that television exposure is a risk factor for overweight in preschoolers (35,36).

Few studies have examined children's media use in child care settings, and more research is needed. A 2006 review of the effects of television viewing by infants and preschoolers found that obesity is largely ignored in the television literature involving preschoolers (37). Children in day care homes spend more time watching television than do children in day care centers (38). Future studies should examine media policies and practices in various child care settings and determine whether high levels of media use are associated with increased sedentary behavior and decreased physical activity levels.

We found that only approximately 20% of states set maximum time limits on media use in at least 1 child care setting. Most of these states limit screen time to a maximum of 2 hours per day. States were more likely to have licensing regulations that defined in a nonquantified manner the appropriate inclusion of media in a child care program of activity; 27% of states provided such direction for providers in at least 1 child care setting.

### Importance of the child care setting

In contrast to the extensive research, policy, and advocacy efforts regarding nutrition and physical activity in the school setting, the child care setting has been largely overlooked in the childhood obesity discussion. Legislative action for nutrition and physical activity in the school setting has been brisk; during 2005, 42 states introduced legislation related to nutrition guidance in schools, and 44 states introduced legislation related to physical activity and education (39). To our knowledge, no similar health policy tracking information exists for child care facilities. Our analysis found relatively little state-level legislative activity related to nutrition, physical activity, or media use in child care facilities.

Child care presents a tremendous opportunity to help children develop healthful nutrition and physical activity attitudes and behaviors. Our study demonstrates that state child care licensing regulations can be improved. Regulatory approaches to nutrition, physical activity, and media use vary widely among states; states with more comprehensive licensing regulations serve as examples for others. Each state should examine its regulatory approaches to the various child care settings and identify opportunities to strengthen regulations. State child care licensing offices can work with public health, nutrition, and physical activity experts to develop regulations that are health-promoting and realistically adoptable in CCCs, LFGHs, and SFHs. Several changes could be made to make state regulations more amenable to childhood obesity prevention and promote healthful habits among children. For example, states that have a general requirement for daily outdoor activity could quantify the minimum amount of time that children should be outdoors, and quantified limits on screen time could be defined for each child care setting. Foods of low nutritional value, such as sugar-sweetened beverages, should be restricted.

State policies need to be strengthened in several areas. However, in making decisions about licensing regulations, a trade-off must be considered. Excessive regulation and administrative burden could result in the unintended consequence of a reduction in the number of family child care home providers who choose to be licensed. Implementing stronger licensing regulations in CCCs, particularly those that are part of large regional or national for-profit chains, may be more feasible.

### Strengths and limitations

This study had several strengths and limitations. We used a methodical, structured, quantitative content analysis of state child care licensing regulations. We determined coding dimensions on the basis of scientific literature and national recommendations for preschool-aged children and child care settings. Intercoder reliability between 2 independent coders indicated strong agreement. However, we could not assess the degree to which child care facilities comply with state regulations. Regulatory enforcement is a challenge in this setting (30). Furthermore, we did not examine policies at the local or individual facility level. Child care facilities under management by particular providers may have adopted additional policies that are stronger and more conducive to healthy nutrition and physical activity, superseding requirements prescribed by the state.

### Conclusions

We demonstrated that state licensing regulations regarding nutrition, physical activity, and media use vary widely among and within states. Nearly three-quarters of US preschool-aged children spend time in nonparental care arrangements each week. State regulations and standards governing nutrition, physical activity, and media use are weak and need to be strengthened. Improvements in these policies could improve the diets and physical activity behaviors of millions of children and improve their health. The increasing prevalence and earlier onset of childhood obesity underscore the urgency of state policy efforts to create child care environments that foster healthy eating and physical activity.

Given the role of child care in the lives of American families, including child care settings in research and policy efforts directed at child health and obesity prevention is essential. State policy makers, health professionals, child care practitioners, and state licensing offices need to work together to develop nutrition, physical activity, and media use policies and regulations that will improve children's health and help prevent obesity, without placing undue administrative or financial burden on child care facilities.

## Figures and Tables

**Table 1:** Benchmarks and Examples for 13 Coding Dimensions Used in Child Care Licensing Regulation Analysis, 50 US States and the District of Columbia, January-April, 2006

**Regulation Benchmarks^a^**	Example From State Licensing Regulations
**Nutrition**	
Meals and snacks should follow federal CACFP or similar meal pattern requirements (12,14,15,17)^b^	"Menus shall comply with the USDA Child and Adult Care Food Program guidelines." (Utah)
Meals and snacks should be consistent with *Dietary Guidelines for Americans* (13,15)	"A center shall provide children with meals and snacks that are consistent with the United States Department of Agriculture's current *Dietary Guidelines for Americans*." (West Virginia)
Specify the proportion of children's daily nutrition needs to be offered per meal or by length of time in care (12,15)	"Each meal must provide one-third of the child's daily nutritional needs as specified by the United States Department of Agriculture, Food and Nutrition Service, in Code of Federal Regulations, title 7, section 226.20." (Minnesota)
Specify no. of meals and snacks to be served in comprehensive increments of time in care, up to a full day (14,17)	"Nutritional and appropriately timed meals and snacks meeting nutritional requirements . . . shall be served in accordance with the following schedule which indicates number of hours child is present at the Center: A. 2 hours to 4 hours, 1 snack. B. 4 hours to 6 hours, 1 meal and 1 snack. C. 7 hours to 11 hours, 2 meals and 1 snack or 2 snacks and 1 meal based on time of child's arrival. D. 12 or more hours, 3 meals and 2 snacks." (Delaware)
Have policy prohibiting or limiting foods of low nutritional value (12-15,17)^c^	"Foods and drinks with little or no nutritional value, i.e. sweets, soft drinks, etc. shall be served only on special occasions and only in addition to the required nutritious meals and snacks." (Georgia)
Have policy on vending machines^d^	"Vending machines shall be prohibited in areas used by the children." (Alabama)
**Physical activity**	
Require that activity program of child care facility provide large muscle or gross motor activity, development, and/or equipment (12,14,27)	"The program of activities shall be planned to provide . . . daily indoor and outdoor activities in which children use both large and small muscles." (Oregon)
Require that children have daily outdoor activity time, weather and health permitting (14)	"Daily supervised outdoor play is required for all children in care, except during inclement or extreme weather or unless otherwise ordered by a health care provider." (New York)
Quantify required minimum length of time for daily outdoor activity^d^	"Daily activities for preschool and school-age children shall include . . . a total of at least one (1) hour of outdoor play for children in attendance a full day unless prevented by weather or special medical reasons." (Missouri)
Specify that children shall be engaged in vigorous or moderate physical activity (13)	"Activities which promote physical development shall include daily opportunities for running, climbing, and other vigorous and varied physical activities."(Hawaii)
Quantify required no. of minutes of physical activity per day or by length of time in care (13,15,27)	"A child care facility shall provide structure and daily activities designed to promote a child's individual physical, social, intellectual, and emotional development. Satisfactory compliance with this subsection requires that . . . opportunities be provided for a minimum of 20 minutes of vigorous physical activity for every 3 hours the facility is open between the hours of 7:00 am and 7:00 pm." (Alaska)
**Media use^e^**	
Define appropriate inclusion of media in child care program of activities, in a nonquantified manner (13,15,34)^f^	"Television viewing, including videos, should not be permitted without the approval of a child's parents, who must be advised of the center's policy regarding television and video viewing." (Colorado)
Quantify maximum amount of time for media use each day or week (34)	"Children will not watch television, videotapes, or play video games for more than one (1) hour a day." (New Mexico)

Abbreviation: CACFP, Child and Adult Care Food Program; USDA, United States Department of Agriculture.

a Benchmarks/references indicate select national organizations that recommend this dimension for young children.

b Also included if they had similar language but did not refer to the federal CACFP by name. In this case, the regulations had to provide guidance for breakfast, lunch, supper, and snacks; use CACFP food components; provide nutrition guidance by defined child age categories; and specify quantities for the various types of foods to be offered to children. This dimension does not indicate providers' formal enrollment in the CACFP.

c The following items were coded: specific foods and beverages of low nutritional value listed (eg, soft drinks, candy); general statements about limiting foods and beverages high in fat, sodium, and sugar; and statements about the permissibility of foods not meeting the state's nutrition regulations for special occasions, such as birthdays and holidays. Examples of specific foods and beverages mentioned in state regulations include sweetened drinks, fruit drinks, soft drinks, imitation milk drinks, carbonated drinks, potato chips, popcorn, Jell-O, candy, cookies, cakes, pies, doughnuts, pastries, croissants, sweets, and desserts.

d No national organizations were identified as having this recommendation.

e Specific types of media mentioned in state regulations include television, digital video discs (DVDs), videocassette recorder (VCR) tapes, movies, computers, computer games, video games, and electronic games.

f This coding dimension captures state regulations in nonquantified terms about the appropriate role of media in a child care program of activity (eg, "media use shall meet a defined educational objective, shall be limited, and shall not be used as a substitute for planned activity").

**Table 2:** State Licensing Regulations Related to Nutrition, Physical Activity, and Media Use for Preschool-Aged Children in Child Care Facilities, 50 US States and the District of Columbia, January-April, 2006^a^

**Regulation**	No. (%) of Child Care Centers, States^b^	No. (%) of Large Family/Group Child Care Homes^c^, States^b^	No. (%) of Small Family Child Care Homes^d^, States^b^
**Nutrition**			
Meals and snacks should follow federal CACFP or similar meal pattern requirements	29 (57) AK, AL, AR, AZ, CA, CT, DE, GA, HI, IA, IL, LA, MD, MN, MO, MS, MT, NC, NH, NM, OH, OK, OR, SC, TN, UT, VA, WI, WV	24 (47) AK, AL, AR, AZ, CT, DE, GA, HI, IA, IL, MA, MI, MN, MO, MS, MT, NH, NM, OH, OK, OR, SC, TN, UT	20 (39) AK, AL, DE, HI, IL, MA, MI, MN, MO, MT, NC, NH, NM, OK, OR, SC, TN, WA, WI, WV
Meals and snacks should be consistent with *Dietary Guidelines for Americans*	2 (4) MI, WV	1 (2) WV	0
Specify proportion of children's daily nutrition needs to be offered per meal or by length of time in care	14 (27) CO, IL, LA, ME, MN, NC, NV, OH, OK, OR, SD, TX, WV, WY	8 (16) CO, IL, NV, OH, OK, SD, TX, WY	6 (12) CO, IL, NV, OK, TX, WY
Specify no. of meals and snacks to be served in comprehensive increments of time in care, up to a full day	19 (37) AZ, CA, CT, DE, HI, IL, KS, MA, MD, MI, MN, MS, NC, NY, OH, TN, TX, WA, WI	11 (22) CT, HI, IL, KS, MS, MT, NE, NY, OH, TN, TX	9 (18) HI, IL, MT, NE, NY, TN, TX, WA, WI
Have policy prohibiting or limiting foods of low nutritional value	12 (24) GA, IA, IL, IN, LA, MS, NC, NJ, NV, OR, TN, WA	7 (14) AZ, GA, MS, NV, OR, TN, WV	4 (8) NV, OR, TN, VT
Have policy on vending machines	4 (8) AL, GA, LA, MS	2 (4) GA, MS	0
**Physical activity**			
Require that activity program of child care facility provide large muscle or gross motor activity, development, and/or equipment	39 (76) AL, AZ, CO, CT, DC, DE, GA, HI, IA, IL, IN, KS, KY, LA, MA, ME, MI, MN, MO, MS, MT, NC, NJ, NV, NY, OH, OK, OR, PA, RI, SD, TN, TX, VA, VT, WA, WI, WV, WY	25 (49) AR, AZ, CO, CT, DE, GA, HI, IA, IL, KY, MA, MI, MN, MO, MS, MT, NV, OH, OK, OR, PA, TX, VT, WV, WY	19 (37) CO, CT, DC, DE, HI, IL, MA, MI, MN, MO, MT, NV, OK, OR, PA, TX, WA, WI, WV
Require that children have daily outdoor activity time, weather and health permitting	36 (71) AK, AL, AR, CO, CT, DC, DE, GA, IL, IN, KS, MA, MD, ME, MI, MN, MO, MS, NC, NE, NJ, NV, NY, OH, OK, OR, PA, RI, SC, TN, TX, UT, VA, WA, WI, WV	27 (53) AK, AL, AR, CO, CT, DE, GA, IL, IN, KS, MA, MI, MO, MS, MT, ND, NV, NY, OH, OR, PA, RI, SC, TN, TX, VA, WV	24 (47) AK, AL, CO, DC, DE, GA, IL, KY, MA, MI, MO, MT, NC, ND, NV, NY, OR, PA, RI, TN, TX, WA, WI, WV
Quantify required minimum length of time for daily outdoor activity	9 (18) AR, DC, GA, KS, ME, MO, MS, VA, WV	5 (10) AR, GA, KS, MO, MS	2 (4) DC, MO
Specify that children shall be engaged in vigorous or moderate^e^ physical activity	8 (16) AK, DE, HI, LA, MD, MT, NC, TN	4 (8) AK, HI, VA, WV	2 (4) AK, HI
Quantify required no. of minutes of physical activity per day or by length of time in care	1 (2) AK	2 (4) AK, MA	2 (4) AK, MA
**Media use**			
Define appropriate inclusion of media in child care program of activities, in a nonquantified manner	11 (22) AL, CO, GA, IL, IN, SC, TN, TX, VT, WI, WV	9 (18) CO, GA, MI, SC, TN, TX, VA, VT, WV	7 (14) CO, GA, MI, TN, TX, WA, WI
Quantify maximum amount of time for media use each day or week	7 (14) AK, GA, ME, MS, NM, TN, VT	9 (18) AK, DE, GA, MI, MS, NM, OR, TN, VT	8 (16) AK, DE, GA, MI, NM, OR, TN, VT

Abbreviation: CACFP, Child and Adult Care Food Program.

a This analysis includes mandatory licensing regulations that pertain to preschool-aged children (typically defined by states as children aged 3 to 5 years) attending child day care centers or homes. Specific regulations for infants, toddlers, school-aged children, residential care, and special needs care are excluded. Regulations from January through April 2006 were downloaded from state Web sites and analyzed.

b Percentages are calculated using 51 as the denominator; analysis includes 50 states and the District of Columbia.

c Typically 7 to 12 children. The District of Columbia, Louisiana, Maine, Maryland, New Jersey, North Carolina, Washington, and Wisconsin do not classify this type of facility and instead have 1 classification of Family Child Care Home; therefore they do not appear in this category. However, Maryland, North Carolina, and Wisconsin allow up to 8 children and Maine and Washington allow up to 12 children in family homes. Family home licensing regulations for these states are reflected in the Small Family Child Care Homes category in this table.

d Typically 6 or fewer children. States with voluntary licensing, certification, or registration for small family child care homes are not included in this analysis.

e No states used the term *moderate* to describe physical activity level.
